# NM23 deficiency promotes metastasis in a UV radiation-induced mouse model of human melanoma

**DOI:** 10.1007/s10585-012-9495-z

**Published:** 2012-06-15

**Authors:** Stuart G. Jarrett, Marian Novak, Nathan Harris, Glenn Merlino, Andrezj Slominski, David M. Kaetzel

**Affiliations:** 1 Department of Molecular and Biomedical Pharmacology, Markey Cancer Center, University of Kentucky College of Medicine, Lexington, KY USA; 2 Present Address: Department of Biochemistry and Molecular Biology, University of Maryland School of Medicine, Baltimore, MD 21201 USA; 3 Laboratory of Cancer Biology and Genetics, National Cancer Institute, Bethesda, MD USA; 4 Department of Pathology and Laboratory Medicine, University of Tennessee Health Science Center, Memphis, TN USA

**Keywords:** Metastasis suppressor, Melanoma, NM23, Ultraviolet radiation, Mutagenesis, DNA repair, Cell motility, Hepatocyte growth factor, Transgenic mice

## Abstract

Cutaneous malignant melanoma is the most lethal form of skin cancer, with 5-year survival rates of <5 % for patients presenting with metastatic disease. Mechanisms underlying metastatic spread of UVR-induced melanoma are not well understood, in part due to a paucity of animal models that accurately recapitulate the disease in its advanced forms. We have employed a transgenic mouse strain harboring a tandem deletion of the *nm23*-*m1* and *nm23*-*m2* genes to assess the combined contribution of these genes to suppression of melanoma metastasis. Crossing of the *nm23*-*h1/nm23*-*h2* knockout in hemizygous-null form ([*m1m2*]^+/−^) to a transgenic mouse strain (hepatocyte growth factor/scatter factor-overexpressing, or HGF^+^ strain) vulnerable to poorly-metastatic, UVR-induced melanomas resulted in UVR-induced melanomas with high metastatic potential. Metastasis to draining lymph nodes was seen in almost all cases of back skin melanomas, while aggressive metastasis to lung, thoracic cavity, liver and bone also occurred. Interestingly, no differences were observed in the invasive characteristics of primary melanomas of HGF^+^ and HGF^+^ × [*m1m2*]^+/−^ strains, with both exhibiting invasion into the dermis and subcutis, indicating factors other than simple invasive activity were responsible for metastasis of HGF^+^ × [*m1m2*]^+/−^ melanomas. Stable cell lines were established from the primary and metastatic melanoma lesions from these mice, with HGF^+^ × [*m1m2*]^+/−^ lines exhibiting increased single cell migration and genomic instability. These studies demonstrate for the first time in vivo a potent metastasis suppressor activity of NM23 in UVR-induced melanoma, and have provided new tools for identifying molecular mechanisms that underlie melanoma metastasis.

**Electronic supplementary material:**

The online version of this article (doi:10.1007/s10585-012-9495-z) contains supplementary material, which is available to authorized users.

## Introduction

Cutaneous malignant melanoma (CMM) represents one of the most aggressive malignancies with its occurrence increasing worldwide at an alarming rate over the past 50 years [1]. Exposure to the ultraviolet radiation (UVR) spectrum in sunlight is well-established as a risk factor for melanoma, with intermittent sun exposure as a child and/or young adult associated with adult-onset malignancy [2, 3]. Currently, few treatment therapies are available for patients with metastatic melanoma, emphasizing the need for better understanding of the mechanisms underlying the disease in its advanced forms.

The first metastasis suppressor gene to be described was *nm23*-*m1* (termed M1 isoform in mouse, H1 in human), initially identified by its diminished expression in metastatic variants of melanoma and breast carcinoma cell lines [4]. In these and a number of other human cancers, low NM23-H1 expression has been associated with increased tumor aggressiveness and poor clinical outcome [5–7]. While NM23 proteins participate in multiple cellular regulatory pathways, mechanism(s) underlying the metastasis suppressor activity of NM23-H1/M1 are not well-understood. The protein displays three distinct enzymatic activities in vitro, including its nucleoside diphosphate kinase (NDPK), histidine kinase and 3′–5′ exonuclease activities [8–10]. The histidine kinase and NDPK have both been suggested to participate in suppression of motile and invasive phenotypes in vitro, and these represent plausible targets of metastasis suppressor function. However, the NDPK and 3′–5′ exonuclease activities also suggest potential roles in DNA repair and/or replication, with NDPK possibly providing balance in nucleotide pools for DNA polymerase activity, and the 3′–5′ exonuclease functioning in proofreading during replication and/or repair. Along these lines, we demonstrated NM23 expression to be critical for genomic stability in the yeast *Saccharomyces cerevisiae* [11]. More recently, we observed important contributions of NM23-H1 and NM23-H2 to maintenance of genomic stability in human melanoma and mouse cell lines, both in basal and genotoxic conditions [12]. Importantly, mice rendered hemizygous-null at the tandemly-arranged *nm23*-*m1* and *nm23*-*m2* loci [*m1m2*]^+/−^ exhibit vulnerability to UVR-induced melanoma in situ and epithelioid cyst formation on tail skin, providing in vivo evidence for the DNA repair function. These observations suggest that loss of *nm23* expression contributes to both the genesis and progression of human melanoma.

To assess the role of NM23-M1 and NM23-M2 in melanoma progression in vivo, we have crossed [*m1m2*]^+/−^ mice with a transgenic mouse strain engineered for overexpression of hepatocyte growth factor (HGF^+^). The HGF^+^ strain exhibits a form of UVR-induced melanoma with human characteristics, rapid growth but minimal metastatic potential [13]. The current study demonstrates that *nm23*-*m1* and *nm23*-*m2* deficiency confers high metastatic potential to melanomas of the HGF^+^ strain, with aggressively-growing metastases occurring at sites analogous to those seen in human melanoma (i.e. lymph node, lung, liver and bone). In addition, cell lines generated from melanomas of the HGF^+^ × [*m1m2*]^+/−^ hybrid exhibit increased motility and genomic instability, implicating functional roles for NM23 proteins in melanoma progression.

## Materials and methods

### UVB/A radiation of mice and melanoma surveillance

Protocols for murine experiments were approved by the Institutional Animal Care and Use Committee at the University of Kentucky (Protocol 00801M2004). Mice were bred and genotyping was performed as described previously [12]. A C57BL/6-derived mouse strain (HGF^+^) overexpressing a melanocyte-targeted HGF transgene under control of the metallothionein promoter [13, 14] was utilized for these studies. Melanocytes in the HGF^+^ strain exhibit a “humanized” distribution of melanocytes within the epidermis, dermis, and epidermal-dermal junction, which renders them more susceptible to DNA damaging effects of UVR [13, 14]. For study of hybrids with another C57BL/6 strain harboring hemizygotic deletion of the *nm23*-*m1* and *nm23*-*m2* loci [*m1m2*]^+/−^ [15], mice were crossed for at least 6 generations. Reduced NM23-M1 and NM23-M2 expression in the HGF^+^ × [*m1m2*]^+/−^ hybrid relative to that of the wild-type C57BL/6 and HGF^+^ strains was verified in skin tissue by immunoblot analysis (Supplemental Fig. 1). Animals from backcrosses were further propagated to maintain the colony. To induce melanoma, neonatal male mice at postnatal day four were subjected to a single erythematous dose of UVB/A radiation (4,000 J/m^2^) in single wells of a 6-well plastic tissue culture plate without the lid. UVR was administered with lamps emitting a spectral output in the 290–400 nm range (72 % UVB, 27 % UVA, < 0.01 % UVC) (UVP, Upland, CA). Irradiated mice displayed skin reddening and occasional superficial desquamation, but without morbidity or mortality. Tumor incidence and growth rate (volume) were monitored weekly, with caliper measurements [16] initiated at first appearance of a raised lesion. Mice were utilized for the study and maintained for 10 months, but were sacrificed earlier if tumors reached a critical size of 500 mm^3^, or if overt signs of tumor-related illness were apparent. Necropsies were performed on all mice, and regional lymph nodes and internal organs were inspected for metastases. All skin lesions were analyzed by standard histopathology and immunohistochemical methods.

### Establishment of murine melanoma cell lines

Melanoma cell cultures were established from melanomas located on back skin arising in both HGF^+^ and HGF^+^ × [*m1m2*]^+/−^ mice using methods previously described [17, 18] (Supplemental Tables 1, 2). Briefly, hair surrounding the melanoma tumor was removed by a chemical depilatory method, followed by sterile excision of the lesion. Tumor tissue was washed with sterile PBS containing Anti–Anti (Invitrogen, Carlsbad, CA), cut with sterile cross blades to yield 1 mm sized pieces, and mixed with collagenase IV solution for 4 h at 4 °C to obtain single cell suspensions. The slurry was then incubated with trypsin–EDTA incubation overnight at 4 °C (Invitrogen, Carlsbad, CA). The resultant cell suspension was collected by centrifugation at 2,000×*g* for 5 min. Cell pellets were resuspended and plated on poly-d-lysine coated cell culture dishes in MCDB media (Sigma, St. Louis, MO) supplemented with 2 mM CaCl_2_, 2.5 μg insulin, cholera toxin (20 nM), TPA (20 nM), stem cell factor (20 nM), 10 % horse serum and 10 % fetal calf serum (Invitrogen) and incubated at 37 °C. Cell lines were maintained in this growth medium for two passages, after which they were maintained in MCDB media. The cell lines established from HGF^+^ were designated A-T1, A-T2 and A-T3, and those from the HGF^+^ × [*m1m2*]^+/−^ named B-T2, B-T5 and B-T6 (Supplemental Tables 1, 2).

### Wound healing (scratch) assays

Cell lines were plated onto 24-well plates (3 × 10^5^ cells/well) and incubated in 5 % CO_2_ at 37 °C and grown to 100 % confluence. The cells were rendered quiescent by changing the medium to MCDB without FBS for 24 h. The wound was achieved by scratching across the center of each well with a 200 μl pipette tip followed by a PBS wash to remove debris. Videomicroscopy (Nixon Eclipse T*i,* Melville, NY) was utilized to monitor cell lines over a 24 h period with images acquired every 30 min. Cell motility was determined by percent wound closure (calculated by dividing distance moved of invading front at the indicated time points) and mean speed of cell migration (calculated by dividing the total distance of migration by indicated time points, at least 20 cells per cell line were analyzed).

### DNA damage and repair assays

Cell lines derived from HGF^+^ and HGF^+^ × [*m1m2*]^+/−^ tumors were irradiated with either 10 J/m^2^ UVB/A following 24 h in reduced serum medium (0.5 %). Removal of pyrimidine (6–4) pyrimidone photoproducts (6–4 photoproducts) was measured by immunoslot blot assay [19]. DNA (100 ng) was isolated (Qiagen, Valencia, CA) heat-denatured at 100 °C for 10 min, applied to a Hybond nitrocellulose membrane (Amersham Biosciences, Piscataway, NJ) using a vacuum-driven slot blot apparatus and fixed by baking for 1 h at 80 °C. Membranes were incubated with mouse monoclonal antibodies specific for 6–4 photoproducts (Cosmo Bio, Tokyo, Japan). Peroxidase-conjugated anti-mouse secondary antibody was used at a dilution of 1/10,000 in blocking buffer. Equal loading of DNA was confirmed by DAPI (Invitrogen, Carlsbad, CA) staining [20].

### Mutation frequency in the *hprt* gene

Acquired resistance of cells to 6-thioguanine (6-TG) is conferred primarily by mutations within the *hprt* locus [21], and quantified as the number of 6-TG-resistant (6-TG^r^) colonies obtained after selection. Frequencies of spontaneous and UVR-induced *hprt* mutations in HGF^+^ and HGF^+^ × [*m1m2*]^+/−^-derived cell lines as described [21]. Cell lines were seeded at 100 cells per well in a 6-well plate, with each line plated with at least six replicates. Cells were exposed to either UVB/A (5 J/m^2^) or sham-treated, then grown in complete MCDB medium supplemented with 20 μM 6-TG [12]. Colonies were counted at 21 days following initial treatment, with colony-forming efficiency derived as the number of 6-TG^r^ colonies as a percentage of initial plating density.

## Results

### A tandem hemizygous deletion of the *nm23*-*m1* and *nm23*-*m2* genes does not affect incidence or growth rate of UVR-induced melanomas in the HGF^+^ mouse

**Fig. 1:**
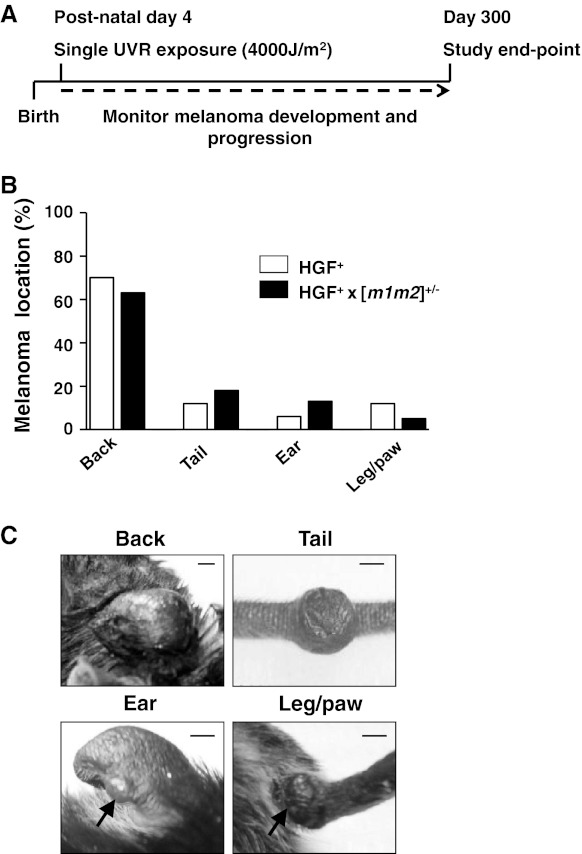
Induction of UVR-induced cutaneous melanoma in HGF^+^ and the HGF^+^ × [*m1m2*]^+/−^ mice. **a** Treatment protocol in which neonatal 4 day old mice were irradiated with 4,000 J/m^2^ UVB/A. **b** Location of UVR-induced cutaneous melanomas. **c** Representative images of tumor locations, *scale bars* represent 5 mm

To assess the potential relationship between NM23 protein expression and melanoma progression, mice harboring a tandem, hemizygotic deletion of the *nm23*-*m1* and *nm23*-*m2* loci ([*m1m2*]^+/−^ strain) [15] were crossed with an HGF-overexpressing strain (HGF^+^). The HGF^+^ strain is prone to UVR-induced melanoma with rapid growth characteristics but low metastatic potential [14, 22]. Newborn litters (post-natal day 4) from the parental strains and the HGF^+^ × [*m1m2*]^+/−^ hybrid were exposed to UVR and monitored for development and growth of skin tumors (Fig. 1a). Tandem, homozygous deletion of the *nm23*-*m1* and *nm23*-*m2* genes is lethal in late embryonic and early neonatal development, preventing analysis of this condition. We have shown previously that UVR treatment of neonatal [*m1m2*]^+/−^ mice induces relatively small nonmetastatic lesions consistent with melanoma in situ on exposed surfaces of the tail, but at no other locations [12]. Confinement of tumors to this location is likely due to the unique external (and UVR-accessible) location of tail melanocytes in the C57BL/6 mouse, which are more deeply sequestered in other skin areas within the dermis and are thus more protected from UVR. In that study, no large melanomas were obtained in the [*m1m2*]^+/−^ strain on other UVR-exposed skin areas such as the back, ears, paws, etc.

**Fig. 2:**
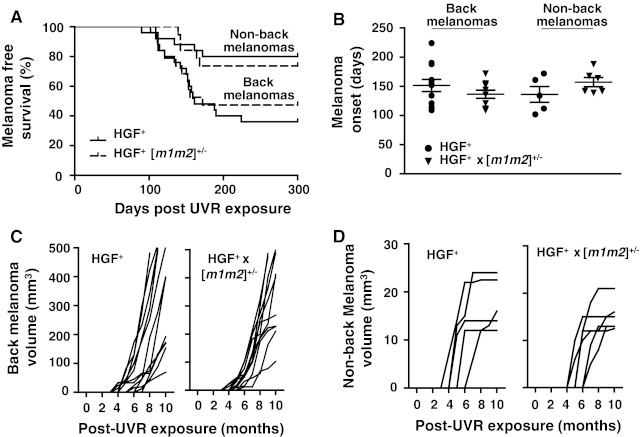
A tandem hemizygous deletion of the *nm23*-*m1* and *nm23*-*m2* genes does not affect UVR-induced incidence or growth rate of melanomas in the HGF^+^ mouse. **a** Kaplan–Meier plot of back and non-back melanoma free survival and **b** mean age of back and non-back melanoma onset (days ± SEM). Tumor growth kinetics of **c** back melanomas (HGF^+^ (*n* = 12); HGF^+^ × [*m1m2*]^+/−^ (*n* = 10) and **d** non-back melanomas [HGF^+^ (*n* = 5)]; HGF^+^ × [*m1m2*]^+/−^ (*n* = 6)

Consistent with prior reports [23, 24], UVR treatment elicited pigmented skin tumors in the majority (68 %) of HGF^+^ mice (Table 1), with no mice displaying more than one tumor. Most of the melanomas were located on the back skin (Fig. 1b, c), although some were also observed on other UVR-exposed locations such as the tail, ear and leg. While time of tumor onset was not significantly different between back and non-back tumors (~150 day; Fig. 2a, b), back tumors grew much more aggressively after their initial appearance (Fig. 2c,d; Supplemental Tables 1, 2).

**Table 1:** UVR-induced melanoma and metastasis in HGF^+^ and HGF^+^ × [m1m2]^+/−^ hybrid mice

Genotype		Back^a^		Tail^b^		Leg/Paw^b^		Ear^b^		Total
*HGF*	*m1m2*	*Incidence*	*Metastasis*	*Incidence*	*Metastasis*	*Incidence*	*Metastasis*	*Incidence*	*Metastasis*	*Incidence*
HGF^+^	WT	12/17(70 %)	0/12(0 %)	2/17(6 %)	0/2(0 %)	1/17(6 %)	0/2(0 %)	2/17(12 %)	0/1(0 %)	17/25(68 %)
HGF^+^	+/−	10/16(63 %)	10/10(100 %)	3/16(18 %)	0/3(0 %)	2/16(13 %)	0/2(0 %)	1/16(5 %)	0/2(0 %)	16/19(84 %)

^a^All back skin melanomas in both groups ranged in size between 100 and 500 mm^3^ at termination of the study

^b^All non-back skin melanomas in both groups were <24 mm^3^ at termination of the study

*Incidence of metastasis from back skin melanomas was significantly different between the groups (*p* < 0.01), as per Mann–Whitney rank sum test

Crossing of the [*m1m2*]^+/−^ genotype into the HGF^+^ background had no effect on melanoma location (Fig. 1b; Table 1), incidence (Fig. 2a, b; Table 1), onset (Fig. 2a, b), grade or volume (Fig. 2c, d; Supplemental Tables 1, 2). The lack of effect of NM23 deficiency on growth of HGF^+^-driven melanomas is consistent with prototypical metastasis suppressor activity, in which metastatic potential but not primary tumor growth per se is impacted. The incidence of lower-grade, UVR-induced pigmented follicular cyst/pigmented epithelioid melanocytoma (PEM) tumors on tail skin was much lower in the HGF^+^ and HGF^+^ × [*m1m2*]^+/−^ hybrid strains than the 100 % penetrance previously reported for the [*m1m2*]^+/−^ strain [12]. This may be attributable to a more intense pigmentation conferred by the HGF^+^ genotype to tail skin of both the HGF^+^ and HGF^+^ [*m1m2*]^+/−^ hybrid strains. In the absence of UVR, no skin tumors were observed in either the HGF^+^ or the HGF^+^ [*m1m2*]^+/−^ hybrid groups, indicating the observed tumors were initiated by UVR treatment.

### *nm23*-*m1* and *nm23*-*m2*-deficiency induces metastasis of UVR-induced melanomas generated on back skin

**Fig. 3:**
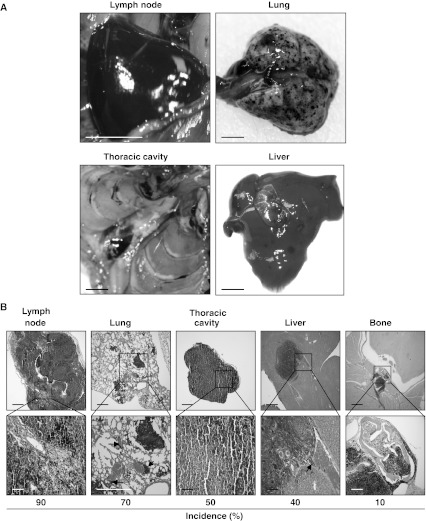
Deficiency in *nm23*-*m1* and *nm23*-*m2*-expression enhances UVR-induced melanoma metastasis. **a** Representative macroscopic appearance of metastases in lymph node, lung, thoracic cavity and liver *scale bar* represent 2 mm. **b** Representative H&E stained sections of common metastatic locations, scale bars represent 500 and 100 μm, respectively. *Arrows* indicate metastases found in proximity to lung and liver blood vessels. *Note* no metastatic dissemination was observed in the HGF^+^ transgenic mouse

To measure the effect of NM23 deficiency on metastasis of UVR-induced melanoma, draining lymph nodes and visceral organs examined both grossly and microscopically for melanocyte infiltration. No macroscopic evidence of metastasis was associated with melanomas arising at any skin location for the HGF^+^ strain, consistent with the low metastatic potential described previously for this model system [22, 25]. In marked contrast, however, all melanomas arising on the back skin of HGF^+^ × [*m1m2*]^+/−^ mice, independent of tumor size and growth rate, showed evidence of metastatic spread (Table 1). No evidence of metastasis was observed with the six slow-growing HGF^+^ × [*m1m2*]^+/−^ melanomas/PEMs that all appeared on skin locations other than the back, which was likely related to their much slower growth and reduced primary tumor mass. Lymph node metastasis was seen in almost all back skin melanomas (9/10), while most also exhibited aggressive lung metastasis (7/10) with numerous pigmented nodules. Most of the affected lungs were also associated with multiple pigmented masses in the thoracic cavity (5/10), with metastases also seen in liver (4/10), and bone (1/10) (Fig. 3a, b; Supplemental Table 2). All of these sites are characteristic of melanoma metastasis in humans. In lymph nodes, metastatic growth was closely associated with node sinuses, strongly suggesting infiltration by melanoma cells occurred via lymphatic vessels (Fig. 3b). Moreover, lung and liver metastases were often seen in proximity to blood vessels, consistent with their entry into those organs via the circulatory system (Fig. 3b). In melanoma-bearing HGF^+^ mice, no microscopic evidence of pigmented micrometastases was evident in potential target organs for metastasis (data not shown).

### Deficiency in *nm23*-*m1* and *nm23*-*m2* expression does not affect histopathological characteristics of UVR-induced primary back melanomas in the HGF^+^ mouse

**Fig. 4:**
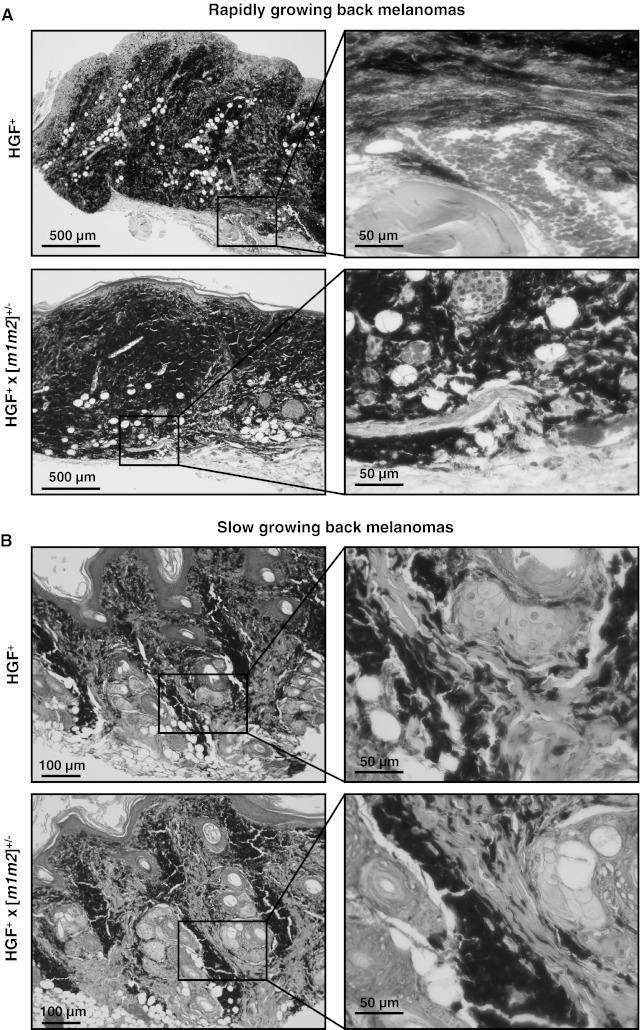
Deficiency in *nm23*-*m1* and *nm23*-*m2* expression does not affect histopathological characteristics of UVR-induced primary back melanomas in the HGF^+^ mouse. Fast and slower-growing melanoma subtypes were observed. Representative H&E-stained sections of **a** rapidly growing back melanoma and **b** slower-growing back melanomas in HGF^+^ and HGF^+^ × [*m1m2*]^+/−^ transgenic animals. *Scale bars* are indicated

UVR-induced melanomas of the HGF^+^ and HGF^+^ × [*m1m2*]^+/−^ strains exhibited histopathological characteristics identical to those previously described for the HGF^+^ strain [26, 27]. The rapidly-growing back skin melanomas consisted of heavily pigmented epitheloid cells, arranged asymmetrically with numerous spindle-shaped cells and mitotic figures (Fig. 4a). In both the HGF^+^ and HGF^+^ × [*m1m2*]^+/−^ groups, heavily pigmented cells filled and expanded the dermis, in most cases extending vertically through the subcutis, reminiscent of that found in CMM human patients [14, 27]. Extensive histological injury to the epidermal and dermal architecture (i.e. ulceration and destruction of hair follicles) was commonly evident. In both mouse groups, a slower-growing melanoma sub-type also was evident and presented with dermal aggregates of heavily pigmented epitheloid cells and subcutical invasion. However, these tumors were characterized by asymmetric vertical strands of tumor tissue, smaller contiguous tumor masses, and minimal intradermal expansion (Fig. 4b). Expression of NM23-M1 and NM23-M2 in primary melanomas of the HGF^+^ × [*m1m2*]^+/−^ group remained at the expected 50 % reduced levels relative to those obtained in HGF^+^ mice (Supplemental Fig. 2). This verified that potential changes, such as compensatory increases or decreases (e.g. due to loss of heterozygosity) did not occur in HGF^+^ × [*m1m2*]^+/−^ tumors and were not a possible underlying cause of their increased metastatic potential. The lack of a clear difference in overall invasive index between back skin melanomas of the two mouse groups, indicates that the high metastatic potential associated with the HGF^+^ × [*m1m2*]^+/−^ genotype is not determined by overall tumor expansion outside the dermal margins.

### Cell lines derived from back skin melanomas of the HGF^+^ × [m1m2]^+/−^ strain exhibit increased cellular motility, impaired DNA repair capacity, and genomic instability

**Fig. 5:**
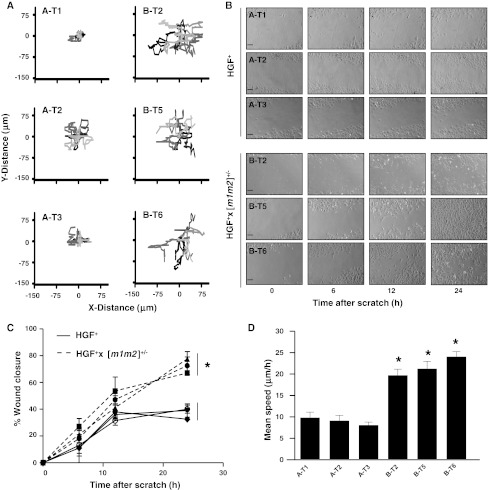
Melanoma cell lines derived from the HGF^+^ × [*m1m2*]^+/−^ transgenic mouse exhibit increased cellular migratory response. Isolated melanoma-derived cell lines were tracked by videomicroscopy over a 24 h time period. **a** Migratory patterns of single cells established from HGF^+^ and HGF^+^ × [*m1m2*]^+/−^ tumors during a 24 h period. **b** Representative photomicrographs of confluent monolayers at 0, 6, 12 and 24 h after wounding in HGF^+^ and HGF^+^ × [*m1m2*]^+/−^ cell lines, *scale bar* represents 20 μm **c** percent closure of wound and **d** mean speed of migration. Values significantly different as determined by 2-way ANOVA (*P ≤ 0.05; *n* = 3; *bars* SEM)

To measure the effect of NM23 expression on motility and genomic stability in melanomas of the HGF^+^ and HGF^+^ × [*m1m2*]^+/−^ mouse strains, cell lines were established for more detailed analysis of these phenotypes. Three cell lines were successfully developed from separate back skin melanomas of the HGF^+^ strain (designated A-T1, A-T2 and A-T3) and HGF^+^ × [*m1m2*]^+/−^ hybrid (B-T2, B-T5 and B-T6) (Supplemental Tables 1, 2). As expected, expression of NM23-M1 and NM23-M2 protein was reduced by approximately 50 % in cell lines established from HGF^+^ × [*m1m2*]^+/−^ melanomas (Supplemental Fig. 3). Spontaneous cell motility was evaluated using the standard scratch/wounding assay in conditions of low mitogenicity culture medium. All three HGF^+^-derived cell lines consisted of cells that displayed minimal migration with short trajectories, in contrast to cells isolated from HGF^+^ × [*m1m2*]^+/−^ melanomas that exhibited highly motile behavior and much longer trajectories (Fig. 5a). Furthermore, the HGF^+^-derived cell lines displayed a slower migration into a “scratch wound” over a 24 h time course, characterized mainly by slow movement of broad adherent cell masses and occasionally single cells (Fig. 5b; Supplemental Video Files 1–6). In addition, to the highly motile nature of HGF^+^ × [*m1m2*]^+/−^ cells, they migrated primarily as detached single cells that travelled at a greater speed which almost completely filled a “scratch” wound within 12–24 h (Fig. 5b, c, d). These results suggest that enhanced single cell migration in NM23-deficient melanoma cell lines may underlie their high metastatic potential.

**Fig. 6:**
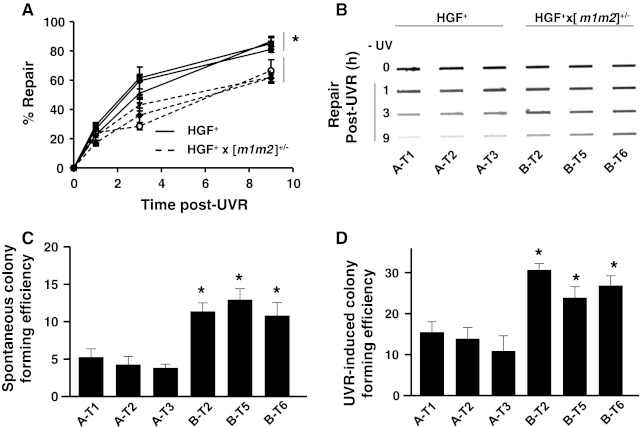
Melanoma cell lines derived from the HGF^+^ × [*m1m2*]^+/−^ transgenic mouse exhibit compromised UVR-induced DNA repair and increased mutagenesis. DNA repair was measured by immuno-slot blot analysis following UVB/A exposure (10 J/m^2^). **a** Relative DNA repair efficiencies and **b** representative immuno-slot blot. **c** Spontaneous and **d** UVR-induced colony-forming efficiency was determined at 21 days post-6-TG treatment as described in “Materials and Methods”. Values significantly different as determined by 2-way ANOVA (*P ≤ 0.05; *n* = 3; *bars* SEM)

To determine whether genomic stability was compromised in back skin melanomas of the HGF^+^ × [*m1m2*]^+/−^ strain, DNA repair capacity and mutation rates were measured in the cell lines derived from each genotype. DNA repair capacity was measured in terms of the elimination of DNA damage in the form of UVB/A-induced 6–4 photoproducts using immuno-slot blot analysis, as previously described [12]. Nucleotide excision repair (NER) is the principal mechanism for repair of UVR-generated (6–4) photoproducts [28], and we recently demonstrated that NER capacity is reduced in *nm23*-deficient murine melanocytes and mouse embryonic fibroblasts [12]. In all three of the HGF^+^ × [*m1m2*]^+/−^ cell lines (6–4) photoproduct repair was slower than that of HGF^+^ cell lines (t_1/2_ 6.3 h vs t_1/2_ 2.5 h; *P* ≤ 0.05) (Fig. 6a, b).

Rates of spontaneous and UVR-induced mutagenesis were quantified in the panel of melanoma cell lines using the 6-TG^r^ colony formation assay [11]. In the absence of UVR exposure, spontaneous mutation rates (i.e. colony formation) were significantly higher in all three HGF^+^ × [*m1m2*]^+/−^ cell lines compared to the HGF^+^ lines (2- to 3-fold; *P* ≤ 0.05) (Fig. 6c). UVB/A exposure caused an overall 2- to 3-fold increase in mutation rates across all six cell lines relative to unexposed cells, with the HGF^+^ × [*m1m2*]^+/−^ cell lines retaining 2-fold higher rates compared to their HGF^+^ counterparts (*P* ≤ 0.05) (Fig. 6d). Taken together, the increases in single cell motility and genomic instability observed in cell lines from NM23-deficient melanomas identifies potential roles for both characteristics in driving the high metastatic potential of these tumors.

## Discussion

The NM23-deficient mouse strain has provided important in vivo validation of the metastasis suppressor function of NM23 in melanoma. Hemizygosity in the *nm23*-*m1* and *nm23*-*m2* genes conferred strong metastatic activity to primary melanomas induced by UVR on back skin in the HGF^+^ mouse strain without effect on primary tumor growth characteristics. This is consistent with prototypical metastasis suppressor activity, and corroborates an earlier report of similar pro-metastatic effects of *nm23*-*m1* deficiency in the context of virus-induced hepatocarcinoma [29]. Our study demonstrates an exquisite sensitivity of the HGF^+^-driven melanoma model, to modest 50 % reductions in NM23-M1 and NM23-M2 expression, with all back skin tumors exhibiting lymph node and/or organ metastasis. Since both the *nm23*-*m1* and *nm23*-*m2* genes were deleted concurrently, it remains to be determined whether one isoform exerts a dominant role or cooperativity exists between them. The Lacombe laboratory [30] has recently reported that silencing *nm23*-*h1*, but not *nm23*-*h2*, promotes the invasive phenotype in liver and colon carcinoma cell lines, strongly suggesting the H1/M1 isoform plays the dominant role in metastasis suppression. Transgenic mice with the *m1* and *m2* genes individually deleted are currently being crossed with the HGF^+^ strain to address this issue directly.

The lack of distinguishing histopathological characteristics associated with primary melanomas of the HGF^+^ × [*m1m2*]^+/−^ strain was surprising in light of the strong pro-metastatic effect of NM23 deficiency. Although poorly metastatic, HGF^+^ melanomas grow aggressively and often invade *en masse* into the subcutical layer of skin, suggesting metastasis is blocked at a later stage of the metastatic cascade (e.g. intravasation, survival in the circulation, extravasation or colonization/angiogenesis). The enhanced ability of HGF^+^ × [*m1m2*]^+/−^- derived melanoma cell lines to migrate autonomously (i.e. in the absence of exogenous chemoattractant) as single cells in scratch assays suggests a clue to the metastatic behavior of these tumors. Such activity could promote migration and invasion toward blood and lymphatic vessels, as well as their traversing of vessel walls at primary and metastatic sites.

While the melanoma-derived cell lines derived from HGF^+^ × [*m1m2*]^+/−^ mice exhibited greater motility/invasion and genomic instability than their HGF^+^-derived counterparts, it remains to be determined whether one or both of these phenotypes are metastasis-suppressing functions. Complementation of the mouse genotype with NM23 mutants that selectively disrupt the motility-suppressing and genome-stabilizing functions of the molecule could offer insights to this key question. Metastasis-prone melanomas of the HGF^+^ × [*m1m2*]^+/−^ strain are likely to have acquired metastasis-driving mutations, and loss of the genomic stabilizing activity of NM23 is a plausible candidate mechanism. NM23 deficiency only impacted the metastatic phenotype and not primary tumor growth (i.e. incidence and melanoma onset), suggesting effects of NM23-dependent genomic instability may be selectively directed to metastasis-driving and not tumor-driving mutations. However, the robust nature of UVR-induced melanomas obtained in the HGF^+^ model strongly suggests tumor-driving mutations are readily acquired independent of NM23 status, which may have obscured the effects of nm23-m1/m2 deficiency on tumor initiation and growth [12]. Taken together, our findings suggest the dual anti-motility and genomic stabilizing activities of NM23 could provide a synergistic boost needed to overcome barriers to metastatic growth.

BRAF mutations at the V600 residue are the predominant MAPK activating mutations found in cutaneous melanoma. Such mutations are rarely if ever seen in the HGF mouse melanoma model [24, 31, 32], which is probably a consequence of constitutive RAF activation downstream of the cell surface receptor for HGF, the MET tyrosine kinase. The ability of NM23 deficiency to confer metastatic potential in HGF-driven melanoma suggests the HGF^+^ × [*m1m2*]^+/−^ strain may be a relevant experimental model for human melanomas that do not harbor BRAF mutations (20–40 %) and are unsuitable candidates for anti-BRAF therapies [33]. Efforts are underway to identify metastasis-driving mutations and other molecular events in NM23-deficient melanomas of these transgenic knockout mouse strains.

## Electronic supplementary material

Below is the link to the electronic supplementary material.

Supplementary material 1 (DOC 25 kb)

Supplementary material 2 (DOC 28 kb)

Supplementary material 3 (DOC 28 kb)

Supplementary material 4 (DOC 23 kb)

Supplementary material 5 (PPT 123 kb)

Supplementary material 6 (PPT 149 kb)

Supplementary material 7 (PPT 143 kb)

Supplementary material 8 (AVI 203687 kb)

Supplementary material 9 (AVI 203687 kb)

Supplementary material 10 (AVI 203687 kb)

Supplementary material 11 (AVI 203687 kb)

Supplementary material 12 (AVI 203687 kb)

Supplementary material 13 (AVI 203687 kb)

## Abbreviations

CMM: Cutaneous malignant melanoma
UVR: Ultraviolet radiation
NDPK: Nucleoside diphosphate kinase
HisK: Histidine kinase
HGF: Hepatocyte growth factor
MAPK: Mitogen-activated protein kinase
NER: Nucleotide excision repair
PEM: Pigmented epithelioid melanocytoma
