# Supplementary material for: NM23 deficiency promotes metastasis in a UV radiation-induced mouse model of human melanoma
Source: Clin Exp Metastasis. 2012 Jun 15;30(1):25–36. doi: 10.1007/s10585-012-9495-z (PMC3547246; doi:10.1007/s10585-012-9495-z)
Supplement: Supplementary file 1 — Supplementary material 1 (DOC 25 kb) [file 10585_2012_9495_MOESM1_ESM.doc]

**Supplemental Table 1** Characteristics of primary melanocytic lesions in HGF+ mice

Primary tumor characteristics

Mouse Tumor Tumor Tumor Tumor Final tumor

No. location classification Reason for termination^b^ onset^c^ growth^d^ volume^e^

A1 Lower back Melanoma 10 month end-point reached 114 56.7 397

A2 Lower back Melanoma Critical tumor mass reached 134 32.4 498

A3 Mid-back Melanoma Tumor ulceration 159 103 481

A4 Mid-back Melanoma Tumor ulceration 152 28.6 172

A5 Mid-back Melanoma 10 month end-point reached 224 56.3 169

A6 Mid-back Melanoma Tumor ulceration 190 93.7 399

A7 Mid-back Melanoma 10 month end-point reached 161 38.4 192

A8 Upper back Melanoma Tumor ulceration 188 33.2 153

A9 Upper back Melanoma Critical tumor mass reached 112 64.6 503

A10 Upper back Melanoma Critical tumor mass reached 109 70.1 491

A11 Upper back Melanoma Tumor ulceration 154 13.8 69

A12 Upper back Melanoma Critical tumor mass reached 121 70.5 511

A13 Tail Melanoma 10 month end-point reached 112 3.2 23

A14 Tail PEM^a^ 10 month end-point reached 134 2.3 14

A15 Ear PEM 10 month end-point reached 161 2.4 12

A16 Ear Melanoma 10 month end-point reached 102 2.4 24

A17 Leg/paw PEM 10 month end-point reached 172 4.0 16

^a^ PEM: pigmented epithelioid melanocytoma

^b^ Primary melanomas were allowed to grow until they reached either/or a volume of 500mm^3^ either/or if signs of illness was apparent

either/or the 10 month post-UVR exposure experimental endpoint was reached

^c^ Tumor onset expressed as days post-UVR exposure

^d^ Estimated tumor volume growth was calculated by dividing final tumor size by the time of growth; expressed as mm^3^ per month

^e^ Final tumor volume expressed in mm^3^

NOTE: Necropsies were performed on all HGF^+^ mice and the draining lymph nodes and visceral organs were investigated for metastasis.

No metastases were observed in the HGF^+^ mouse.
