# Supplementary material for: NM23 deficiency promotes metastasis in a UV radiation-induced mouse model of human melanoma
Source: Clin Exp Metastasis. 2012 Jun 15;30(1):25–36. doi: 10.1007/s10585-012-9495-z (PMC3547246; doi:10.1007/s10585-012-9495-z)
Supplement: Supplementary file 2 — Supplementary material 2 (DOC 28 kb) [file 10585_2012_9495_MOESM2_ESM.doc]

**Supplemental Table 2** Characteristics of primary melanocytic and metastatic lesions in HGF^+^ x [*m1m2*]^+/-^ mice

Primary tumor characteristics Sites of metastasis

Mouse Tumor Tumor Tumor Tumor Final tumor Lymph Thoracic

No. location classification Reason for termination^b^ onset^c^ growth^d^ volume^e^  node Lung cavity Liver Bone

B1 Lower back Melanoma Critical tumor mass reached 144 87.4 487 X X

B2 Lower back Melanoma Tumor ulceration 152 20.4 102 X X X

B3 Lower back Melanoma Tumor ulceration 136 37.8 227 X

B4 Mid-back Melanoma Critical tumor mass reached 121 81.5 489 X X X X

B5 Upper back Melanoma Tumor ulceration 112 32.5 228 X X X

B6 Upper back Melanoma Tumor ulceration 172 42.0 210 X X X X

B7 Upper back Melanoma Weight loss/shortness of breath 153 44.5 267 X X X X X

B8 Upper back Melanoma Critical tumor mass reached 111 79.0 474 X

B9 Upper back Melanoma Critical tumor mass reached 109 71.5 501 X

B10 Upper back/ Melanoma Critical tumor mass reached 155 68.6 412 X

neck

B11 Tail Follicular cyst/ 10 month end-point reached 142 2.0 12

PEM^a^

B12 Tail Follicular cyst/ 10 month end-point reached 168 4.0 21

PEM

B13 Tail PEM 10 month end-point reached 164 3.0 15

B14 Ear PEM 10 month end-point reached 142 3.0 13

B15 Leg/paw Melanoma 10 month end-point reached 188 3.0 13

B16 Leg/paw PEM 10 month end-point reached 139 3.0 16

^a^ PEM: pigmented epithelioid melanocytoma

^b^ Primary melanomas were allowed to grow until they reached either/or a volume of 500mm^3^ either/or if signs of illness was apparent

either/or the 10 month post-UVR exposure experimental endpoint was reached

^c^ Tumor onset expressed as days post-UVR exposure

^d^ Estimated tumor volume growth was calculated by dividing final tumor size by the time of growth; expressed as mm^3^ per month

^e^ Final tumor volume expressed in mm^3^

NOTE: Necropsies were performed on all HGF^+^ x [m1m2]^+/-^ mice and the draining lymph nodes and visceral organs were investigated for metastasis.
