# Supplementary material for: NM23 deficiency promotes metastasis in a UV radiation-induced mouse model of human melanoma
Source: Clin Exp Metastasis. 2012 Jun 15;30(1):25–36. doi: 10.1007/s10585-012-9495-z (PMC3547246; doi:10.1007/s10585-012-9495-z)
Supplement: Supplementary file 3 — Supplementary material 3 (DOC 28 kb) [file 10585_2012_9495_MOESM3_ESM.doc]

**Supplemental Table 3.** NM23-M1 and -M2 protein levels in the primary tumor mass were not altered to that of matched

unaffected skin in both the HGF^+^ and HGF^+^ x [*m1m2*]^+/-^ groups.

Tissue NM23-M1 levels Tissue NM23-M2 levels

(relative to β-tubulin)^b^ (relative to β-tubulin)^b^

Tumor M1/M2 Tumor

No^a^. genotype classification Metastatic Normal Melanoma Normal Melanoma

AT-3 WT Melanoma No 1.4 1.5 0.8 1.0

AT-5 WT Melanoma No 1.4 1.4 0.9 1.1

AT-8 WT Melanoma No 1.0 0.9 0.7 1.6

(X: 1.2 ± 0.2) (X: 1.2 ± 0.3) (X: 0.8 ± 0.1) (X: 1.0 ± 0.1)

BT-2 +/- Melanoma Yes 0.6 0.6 0.4 0.4

BT-5 +/- Melanoma Yes 0.4 0.4 0.3 0.4

BT-10 +/- Melanoma Yes 0.3 0.3 0.3 0.2

(X: 0.43 ± 0.15) (X: 0.43 ± 0.15) (X: 0.33 ± 0.05) (X: 0.33 ± 0.1)

^a^ For information on tumor characteristics see supplemental tables 1 and 2.

^b^ Protein levels of NM23 are expressed as arbitrary units determined by densitometry analysis.
