# Supplementary material for: NM23 deficiency promotes metastasis in a UV radiation-induced mouse model of human melanoma
Source: Clin Exp Metastasis. 2012 Jun 15;30(1):25–36. doi: 10.1007/s10585-012-9495-z (PMC3547246; doi:10.1007/s10585-012-9495-z)
Supplement: Supplementary file 4 — Supplementary material 4 (DOC 23 kb) [file 10585_2012_9495_MOESM4_ESM.doc]

**Supplemental Figure Legends**

**Supplemental Figure 1.** Confirmation of NM23-M1 and NM23-M2 protein expression in HGF^+^ and HGF^+^ x [*m1m2*]^+/-^ mice. Relative NM23 protein levels were determined from skin biopsies via immuno-blot. For comparison, the parental strains of C57BL/6 and C57BL/6 x [*m1m2*]^+/-^ were included.

**Supplemental Figure 2.** NM23-M1 and -M2 protein levels in the primary tumor mass were not altered to that of matched unaffected skin in both the HGF^+^ and HGF^+^ x [*m1m2*]^+/-^ groups. Relative NM23 protein levels were determined from skin biopsies taken from the primary melanoma and adjacent normal skin in **(a)** HGF^+^ and **(b)** HGF^+^ x [*m1m2*]^+/-^ mice. Representative immuno-blots displaying NM23 levels are shown relative to β-tubulin.

**Supplemental Figure 3.** Confirmation of NM23-M1 and NM23-M2 protein expression in cell lines established from HGF^+^ and HGF^+^ x [*m1m2*]^+/-^ melanomas. Representative immuno-blots displaying NM23 levels in HGF^+^ (AT-1, AT-2 and At-3) and HGF^+^ x [*m1m2*]^+/-^ (BT-2, BT-5 and BT-6) generated cell lines are shown relative to β-tubulin.
