# Supplementary figures and images for: NM23 deficiency promotes metastasis in a UV radiation-induced mouse model of human melanoma
Source: Clin Exp Metastasis. 2012 Jun 15;30(1):25–36. doi: 10.1007/s10585-012-9495-z (PMC3547246; doi:10.1007/s10585-012-9495-z)

## Slide 1
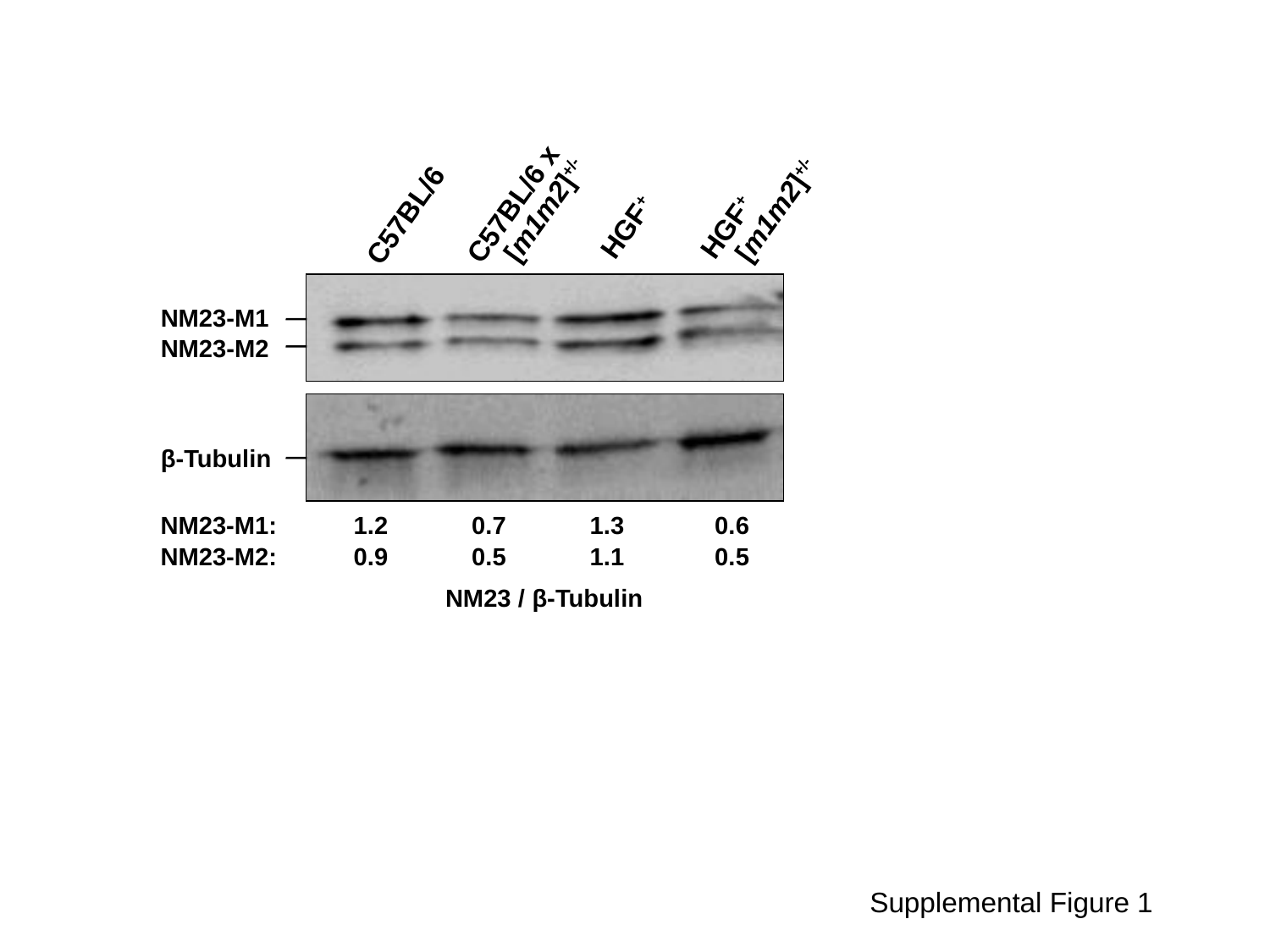

C57BL/6 x
[m1m2]+/-
[m1m2]+/-
C57BL/6
HGF+
HGF+
NM23-M1
NM23-M2
β-Tubulin
NM23-M1: 1.2 0.7 1.3 0.6
NM23-M2: 0.9 0.5 1.1 0.5
NM23 / β-Tubulin
Supplemental Figure 1

Supplement: Supplementary file 5 — Supplementary material 5 (PPT 123 kb) [file 10585_2012_9495_MOESM5_ESM.ppt]

## Slide 1
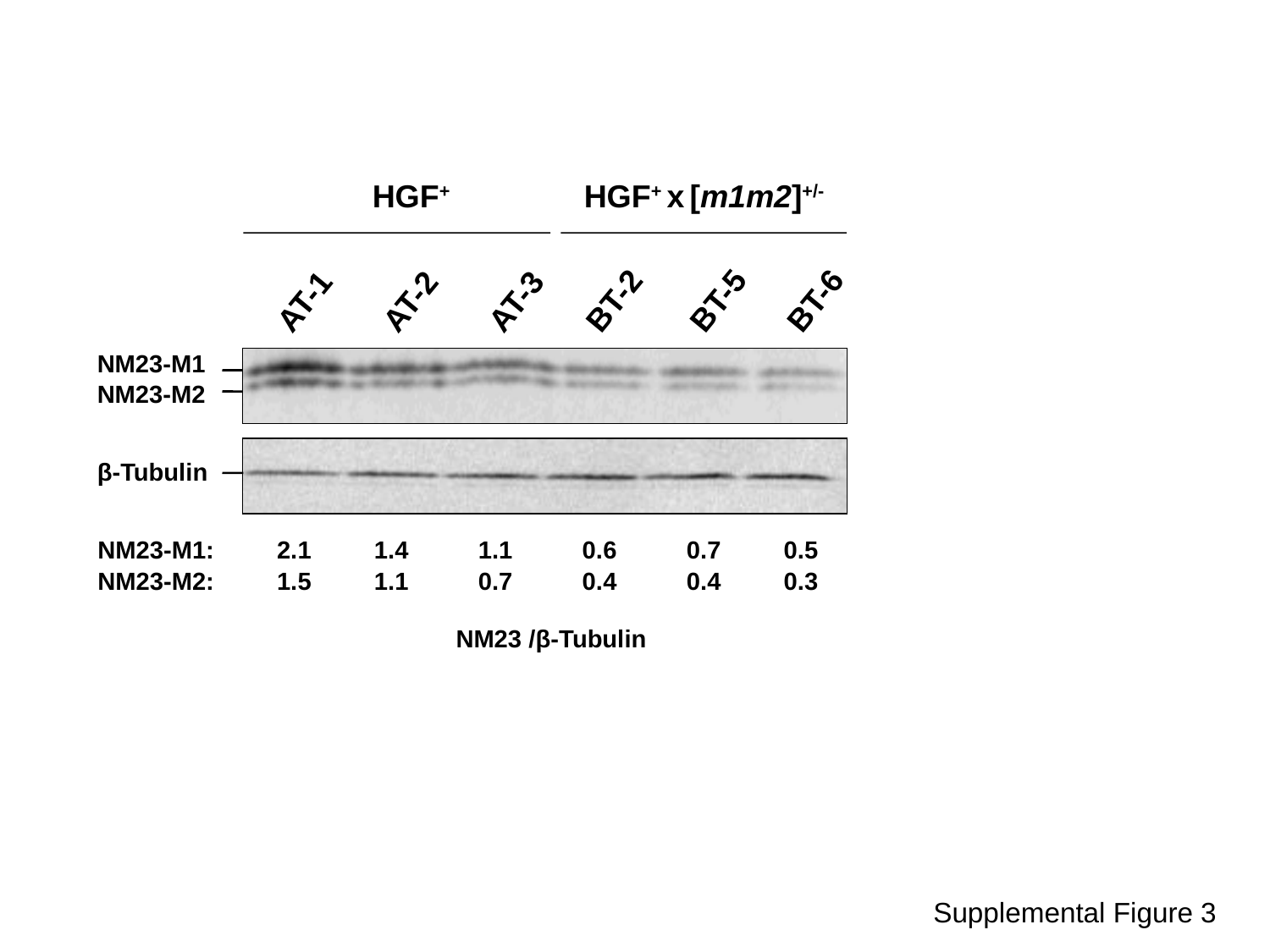

HGF+
HGF+ x [m1m2]+/-
AT-1
AT-2
AT-3
BT-2
BT-5
BT-6
NM23-M1
NM23-M2
β-Tubulin
NM23-M1: 2.1 1.4 1.1 0.6 0.7 0.5
NM23-M2: 1.5 1.1 0.7 0.4 0.4 0.3
NM23 /β-Tubulin
Supplemental Figure 3

Supplement: Supplementary file 7 — Supplementary material 7 (PPT 143 kb) [file 10585_2012_9495_MOESM7_ESM.ppt]
