# Supplementary material for: NM23 deficiency promotes metastasis in a UV radiation-induced mouse model of human melanoma
Source: Clin Exp Metastasis. 2012 Jun 15;30(1):25–36. doi: 10.1007/s10585-012-9495-z (PMC3547246; doi:10.1007/s10585-012-9495-z)
Supplement: Supplementary file 6 — Supplementary material 6 (PPT 149 kb) [file 10585_2012_9495_MOESM6_ESM.ppt]

## Slide 1
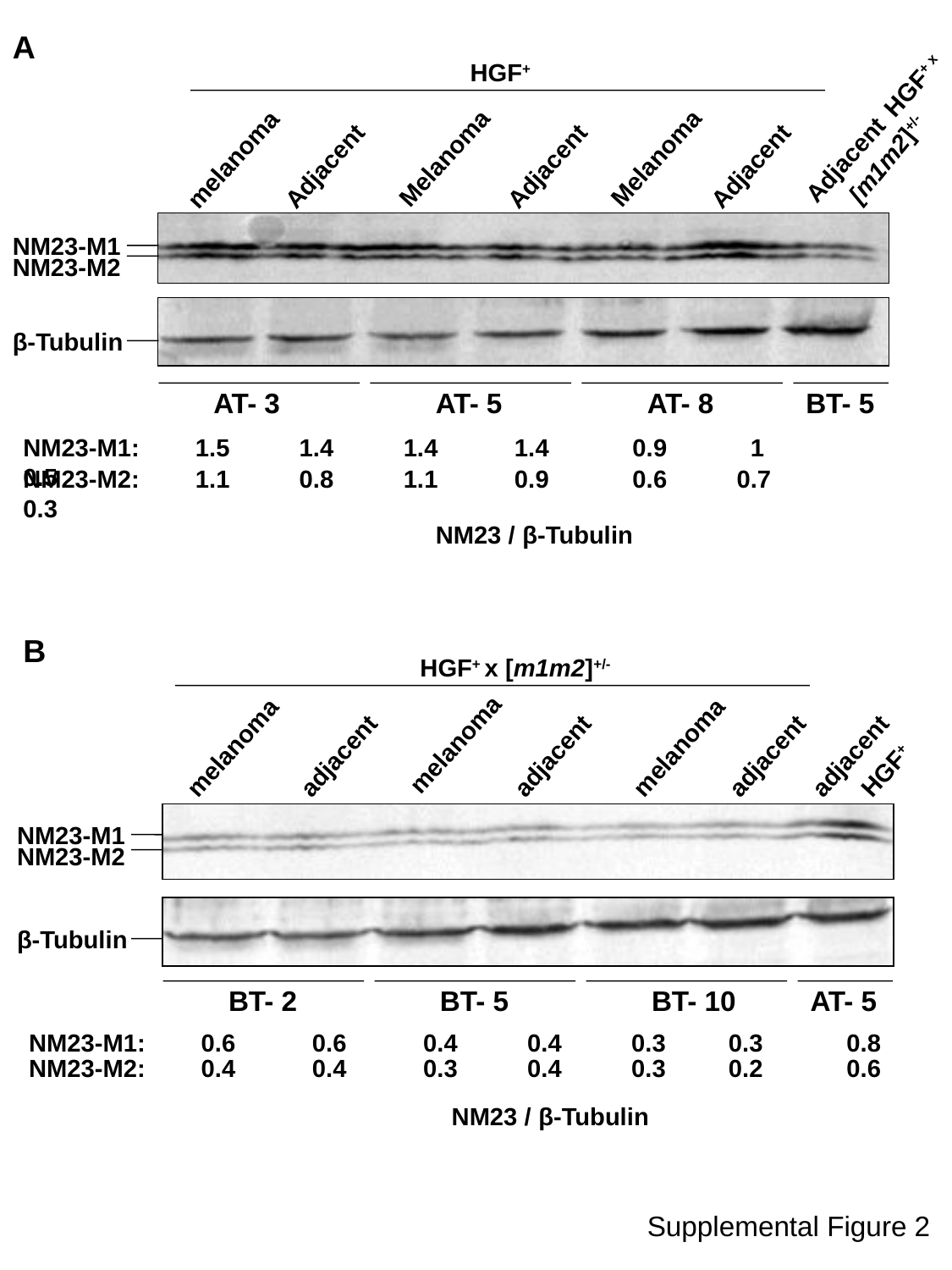

A
HGF+
HGF+ x
Melanoma
Melanoma
melanoma
Adjacent
Adjacent
Adjacent
[m1m2]+/-
Adjacent
NM23-M1
NM23-M2
β-Tubulin
AT- 3
AT- 5
AT- 8
BT- 5
NM23-M1: 1.5 1.4 1.4 1.4 0.9 1 0.5
NM23-M2: 1.1 0.8 1.1 0.9 0.6 0.7 0.3
NM23 / β-Tubulin
B
HGF+ x [m1m2]+/-
melanoma
melanoma
melanoma
adjacent
adjacent
adjacent
adjacent
HGF+
NM23-M1
NM23-M2
β-Tubulin
BT- 2
BT- 5
BT- 10
AT- 5
NM23-M1: 0.6 0.6 0.4 0.4 0.3 0.3 0.8
NM23-M2: 0.4 0.4 0.3 0.4 0.3 0.2 0.6
NM23 / β-Tubulin
Supplemental Figure 2
